# Graphene‐Nanowall‐Decorated Carbon Felt with Excellent Electrochemical Activity Toward VO_2_^+^/VO^2+^ Couple for All Vanadium Redox Flow Battery

**DOI:** 10.1002/advs.201500276

**Published:** 2015-12-31

**Authors:** Wenyue Li, Zhenyu Zhang, Yongbing Tang, Haidong Bian, Tsz‐Wai Ng, Wenjun Zhang, Chun‐Sing Lee

**Affiliations:** ^1^ Functional Thin Films Research CentreShenzhen Institute of Advanced TechnologyChinese Academy of SciencesShenzhen518055P. R. China; ^2^ Department of Physics and Materials ScienceCenter of Super‐Diamond and Advanced Films (COSDAF)The City University of Hong KongHong Kong SARP. R. China

**Keywords:** 3D, electrochemical activity, energy storage, graphene nanowalls, vanadium redox flow battery

## Abstract

3D graphene‐nanowall‐decorated carbon felts (CF) are synthesized via an in situ microwave plasma enhanced chemical vapor deposition method and used as positive electrode for vanadium redox flow battery (VRFB). The carbon fibers in CF are successfully wrapped by vertically grown graphene nanowalls, which not only increase the electrode specific area, but also expose a high density of sharp graphene edges with good catalytic activities to the vanadium ions. As a result, the VRFB with this novel electrode shows three times higher reaction rate toward VO_2_^+^/VO^2+^ redox couple and 11% increased energy efficiency over VRFB with an unmodified CF electrode. Moreover, this designed architecture shows excellent stability in the battery operation. After 100 charging–discharging cycles, the electrode not only shows no observable morphology change, it can also be reused in another battery and practical with the same performance. It is believed that this novel structure including the synthesis procedure will provide a new developing direction for the VRFB electrode.

## 1 Introduction

Vanadium redox flow battery (VRFB) has attracted wide attention for its merits of high capacity, fast response time, tolerance to over‐loading and low maintenance which make it an ideal energy storage solution for loading shifting in power grid as well as in wind and solar energy conversion systems.^1^, ^2^, ^3^ Despite the great promise of VRFB, many challenges need to be addressed before they can find wider applications.^4^ One of the toughest problems is the low electrochemical activity of their electrodes (typically carbon or graphite felts) which decrease the battery's energy efficiencies significantly.^5^, ^6^, ^7^, ^8^, ^9^, ^10^ In contrast to the electrodes of conventional lithium ion batteries that convert electric energy to chemical energy via lithium ion insertion/extraction processes, the electrodes of VRFBs only provide active surfaces (sites) for redox reactions without being involved in them.^11^ So the most effective way to enhance the electrochemical activity of electrodes is to increase their surface area and thus the number of active sites on them. Moreover, compared with the negative redox reactions (V^3+^ + e^−^ = V^2+^, only one electron transfer), the positive redox reactions (VO_2_^+^ + 2H^+^ + e^−^ = VO^2+^ + H_2_O) involve at least three elementary steps (one electron transfer and two proton exchanges), which make kinetics of the VO_2_^+^/VO^2+^ reaction much slower.^12^ Therefore, the electrochemical kinetics of VRFB are limited by the positive reactions, leading to the significance of developing positive electrodes with high electrochemical activity toward VO_2_^+^/VO^2+^ redox processes.^13^, ^14^, ^15^

Over the past few years, intensive efforts have been devoted to address the above‐mentioned challenge by modifying the electrode surface to introduce more active sites or to enhance their catalytic activities. For example, noble metals such as Au, Pt, Ru, and Ir have been deposited on the carbon felt (CF) electrodes and exhibited improved catalytic activity toward VO_2_^+^/VO^2+^ redox couple. However, in addition to the high cost of these metals, they are also active toward water splitting. As a result, electrical energy will be wasted on the undesirable water splitting process instead of the redox reactions of vanadium ions. This not only decreases the energy efficiency (EE), but also leads to oxygen evolutions which in turn causes stability and safe problem in the battery.^16^, ^17^ Recently, some low cost metal or metal oxides were successfully introduced on CFs though hydrothermal or electrochemical deposition method, which shows better electrochemical performance than noble metals, especially at high charge–discharge current density.^18^, ^19^, ^20^, ^21^ For example, Li et al. have successfully demonstrated low cost bismuth and niobium oxide nanoparticles modified CF electrodes, which can effectively reduce the electrochemical polarization of the electrode during charge–discharge processes.^22^, ^23^

On the other hand, graphene materials have also been found to have promising applications on electrodes for VRFB.^24^, ^25^, ^26^ First, the excellent conductivity of graphene materials would decrease the contact resistance and facilitate efficient electron transfer during the electrode reactions. Second, their large specific surface areas and easy surface functionalization can also provide considerably increase in active reaction sites (oxygen containing functional groups) for vanadium ions. Third, the graphene materials are inert to the strongly acidic electrolyte and thus avoiding contamination of the electrolyte by the metal oxides' dissolving products.^3^ González et al. have prepared carbon nanowalls thin films on gold substrate and analyzed their catalytic activity toward VO_2_^+^/VO^2+^ couple, these thin films show good electrochemical activity ascribing to their high specific area and oxygen containing groups on them.^27^ Han et al. have electrosprayed simultaneously carbon nanotube and reduced graphene oxide onto a glassy carbon disk to prepare an electrode with enhanced electrocatalytic performance toward the positive VO_2_^+^/VO^2+^ couple.^28^ By soaking CF in a dispersion of functionalized graphene nanoplatelets, Park et al. have demonstrated an electrode with superior electrochemical activity toward vanadium ion couples.^29^ On top of these promising results, it is expected that there is still much room for further performance enhancement via (1) avoiding stacking of the graphene material which limits the effective area of the electrodes and (2) improving the bonding between the graphene and the electrode for enhancing the system's stability.

To achieve these improvements, we designed and prepared a unique hierarchical structure by growing a uniform and dense layer of 3D graphene on CF via a simple microwave plasma enhanced chemical vapor deposition (MPCVD) process. It was observed that the carbon fibers in CF were successfully wrapped by vertically grown graphene nanowalls, which not only increase the electrode specific area, but also exposing a high density of sharp graphene edges with good catalytic activities to the vanadium ions. We applied the electrode in VRFB and observed about three times higher reaction rate toward VO_2_^+^/VO^2+^ redox couple and 11% increased EE over VRFB with an unmodified CF electrode. It was also found that the graphene‐coated CF electrode show excellent stability in the battery operation. After 100 charging–discharging cycles, the electrode not only shows no observable morphology changes, it can also be reused in another battery and practically with the same performance.

## 2 Results and Discussion

**Figure 1:**
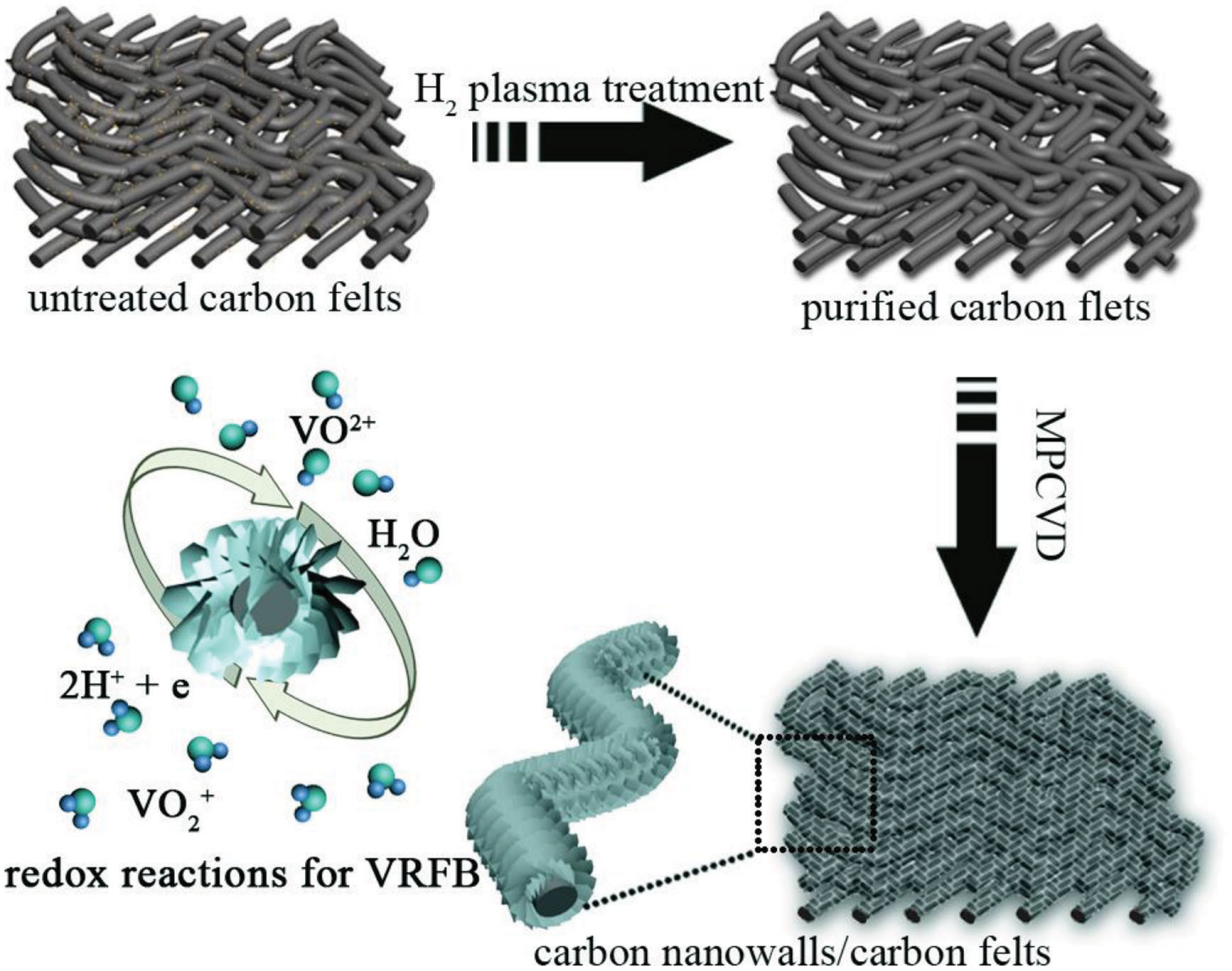
Illustration of fabrication process and the designed structure of 3D graphene‐nanowall‐modified CF.

**Figure** **1** shows schematically the preparation process for the graphene‐nanowall‐modified CF (CF‐G). CF, a commonly used electrode for VRFB, is comprised of cross‐linked carbon fibers with diameters form several to tens micrometers. Pristine CF was first cleaned with a hydrogen plasma for 10 min to remove contamination. 3D graphene nanowalls were then grown onto carbon fibers of the CF by MPCVD (experimental parameters are summarized in Table S1, Supporting Information).

**Figure 2:**
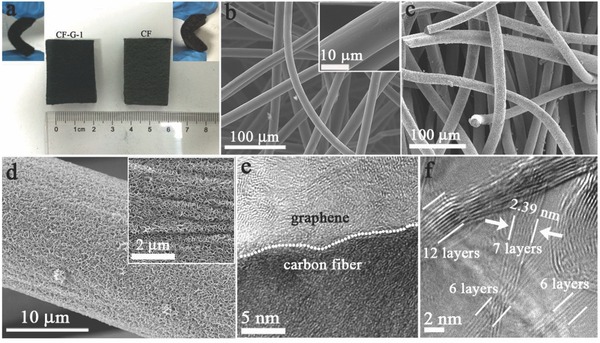
a) A typical photograph of the as‐prepared graphene‐nanowall‐decorated CF (CF‐G‐1) and pristine CF. b) SEM images of CF and c.d) CF‐G‐1 under different magnification and e,f) HRTEM images for CF‐G‐1.

A typical photograph of an as‐prepared electrode material (CF‐G‐1) is shown in **Figure** **2**a. Except that the surface color of the graphene modified CF becomes darker than the pristine CF, little change can be observed. The pristine CF used in here contains carbon fibers of 15 μm diameter with smooth surface (Figure 2b). After the MPCVD process, the carbon fibers are successfully wrapped by vertically standing graphene nanowalls, with the diameter increasing to 20–30 μm (Figure 2c,d). It was found that the density and the morphology of the nanowalls can also be easily controlled by changing the ratio of reactant gases and deposition time (Figure S1, Supporting Information). Specific surface area (S) of the electrodes increase significantly upon graphene deposition. For example, the S value for CF‐G‐1 is about 11.8 m^2^ g^−1^ while that of CF is only about 1.9 m^2^ g^−1^ (Figure S2, Supporting Information). In addition, compared with bare CF sample, all CF‐G samples show significantly decreased area electric resistance after the 3D graphene in situ growth process (Figure S3, Supporting Information), implying smaller physical polarization for CF‐G electrodes during the VRFB operation.

The CF‐G‐1 material was grinded into powder, dispersed in alcohol solution and dropped onto a porous carbon film for transmission electron microscopy (TEM) observation. Figure 2e shows an interface region between a carbon fiber and the graphene nanowall. It can be seen that the interface is clean and shows no observable transition layer hinting a good bonding between the materials. Figure 2f shows that most of the graphene walls consist of about 6–10 graphene layers.

**Figure 3:**
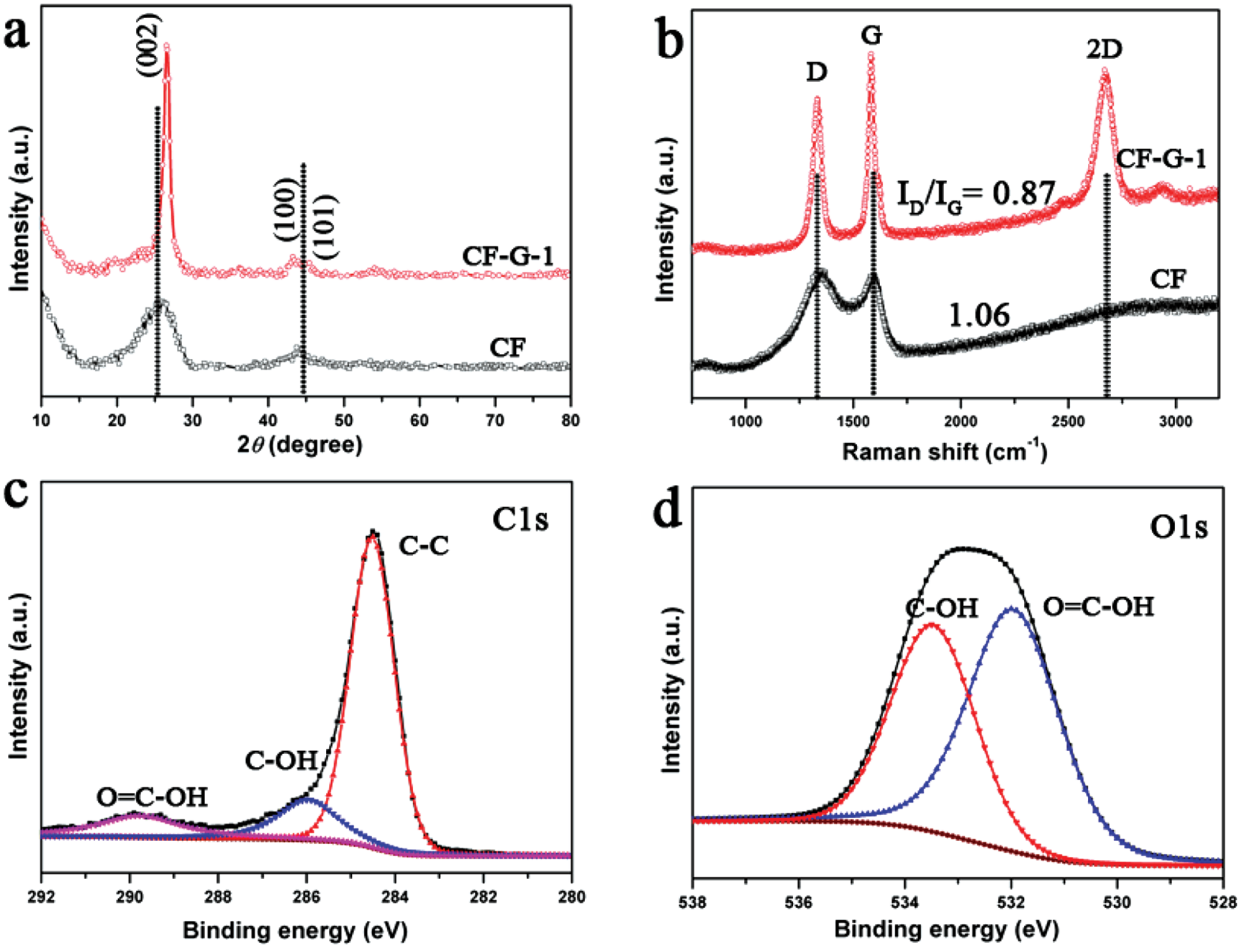
XRD patterns a) and Raman spectra b) of CF‐G‐1 and CF materials. c) XPS C1s and d) O1s high‐resolution spectra for CF‐G‐1 materials.

Crystal structures of the CF and the CF‐G‐1 samples were characterized with X‐ray diffraction (XRD). As shown in **Figure** **3**a, the two diffraction peaks at 26.5° and 44.0° can be assigned to the (002) and the (100) planes of graphite.^30^ After the deposition process, the (002) peak intensity of CF‐G‐1 becomes much stronger than that of CF, suggesting the well‐ordered structure of the vertically nanowalls. Raman spectroscopy was used to provide more information on structural properties of the obtained vertically nanowalls decorated CF materials (Figure 3b). The enhancement in Raman signal at the 1584 cm^−1^ (G band) and the appearance of shape peak at 2668 cm^−1^ (2D band) confirming the TEM observation that the vertically nanowalls are several layer graphene materials.^31^, ^32^ These characteristics can also be observed for other samples of CF‐G materials (Figure S4, Supporting Information). To better understand the oxygen containing functional groups on the surface of graphene nanowalls, we employed X‐ray photoelectron spectroscopy (XPS) analysis. For the CF‐G‐1 sample, the high‐resolution C1s region displays three carbon peaks corresponding to C—C, C—OH, and O—C—OH bonds (Figure 3c).^29^ As shown in Figure 3d, the O1s peak of the CF‐G‐1 assigned to 531.5 and 533.2 eV can be attributed to C—OH and C=C—OH bond, respectively, consistent with the C1s analysis result. The detailed elemental compositions for CF‐G samples are presented in Figure S5 of the Supporting Information. These oxygen containing functional groups will provide effective reaction sites for VO_2_^+^/VO^2+^ redox reactions.^29^

**Figure 4:**
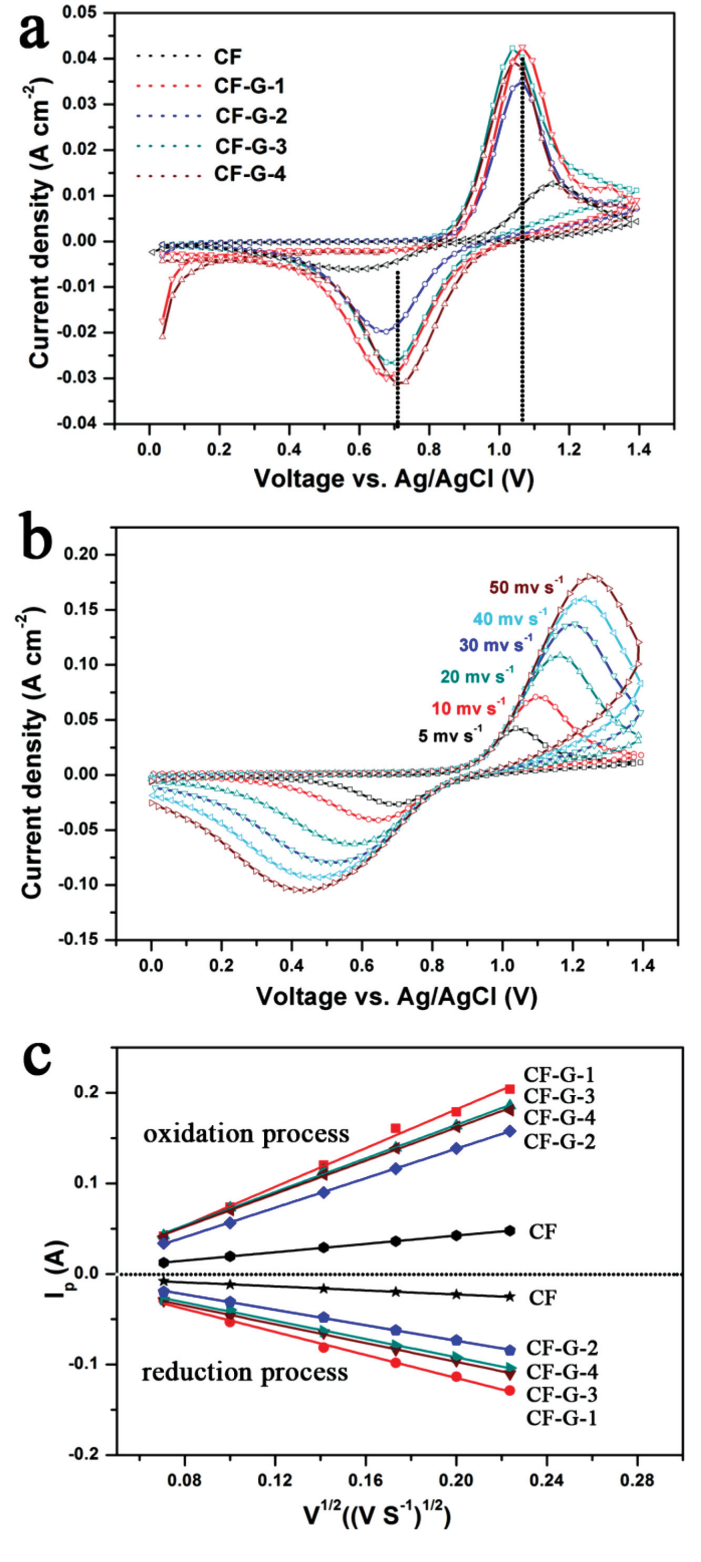
a) CV comparison of the CF, CF‐G‐1, CF‐G‐2, CF‐G‐3, and CF‐G‐4 at a scan rate of 5 mV s^−1^. b) CV curves of CF‐G‐1 electrodes under different scan rates range from 5 to 50 mV s^−1^. c) Plot of the anodic (Ipa) and cathodic (Ipc) peak current versus the square root of each scan rate for different samples.

**Figure** **4**a shows cyclic voltammetry (CV) curves for different electrodes obtained at a scanning rate of 5 mV s^−1^ (the values of peak current, peak potential and peak potential difference are listed in Table S2, Supporting Information). The peaks at about 1.1 V correspond to the oxidation process of VO^2+^ → VO_2_^+^, with the oxygen transferring from water to VO^2+^ and electron output (this process is also presented in Figure 1).^33^, ^34^ Accordingly, the peaks at about 0.75 V can be assigned to the reduction process of VO_2_^+^ → VO^2+^.^35^ Peak current, peak potential difference as well as the redox onset potentials can be used to estimate the catalytic activity of electrode materials.^36^ Obviously, the peak currents for the graphene‐nanowall‐decorated CF electrodes (CF‐G‐1, 2, 3, and 4) are about three to four times the value obtained on the pristine CF electrode, and the redox onset potentials and peak potential difference values of the pristine CF electrode are much larger than those for the CF‐G electrodes, implying considerably improved catalytic activity of the CF‐G electrodes. CV curves of the CF‐G‐1 and the CF electrodes were measured at different scanning rates in 0.1 m VOSO_4_ + 2 m H_2_SO_4_ solution are exhibited, respectively, in Figure 4b and Figure S6 of the Supporting Information. Corresponding data for the other CF‐G electrodes are shown in Figure S7a–c of the Supporting Information. These oxidation/reduction processes on all the electrodes studied here show the same features that both the peak currents and the peak potential difference values increase with the scan rate, indicating these electrode processes are irreversible.^37^ The mass transfer properties can be assessed by plotting the peak current density versus the square root of scan rate from the Randles–Sevcik equation.^38^ The good linearity of the plots as shown in Figure 4c indicates that a diffusion process controls the vanadium redox reaction on the surface of graphene nanowalls.^39^ In addition, the slopes of the graphene‐nanowall‐decorated CF electrode are larger than that of CF electrode samples, suggesting that a faster mass transfer process could be achieved on the graphene‐nanowall‐modified electrode.^11^ In order to get more accurate values to compared all these electrode, reaction rate constant (K°) for all oxidation and reduction reactions are calculated (details are described in the Supporting Information, Figure S7d–f, Equations (S1) and (S2), and Table S3).^40^ The K° values for oxidation and reduction processes on the CF electrode are only 1.84 × 10^−3^ and 2.43 × 10^−3^ cm s^−1^, respectively, which are much smaller than those of corresponding values on the CF‐G electrodes (e.g., 5.79 × 10^−3^ and 7.20 × 10^−3^ cm s^−1^ for CF‐G‐1).

**Figure 5:**
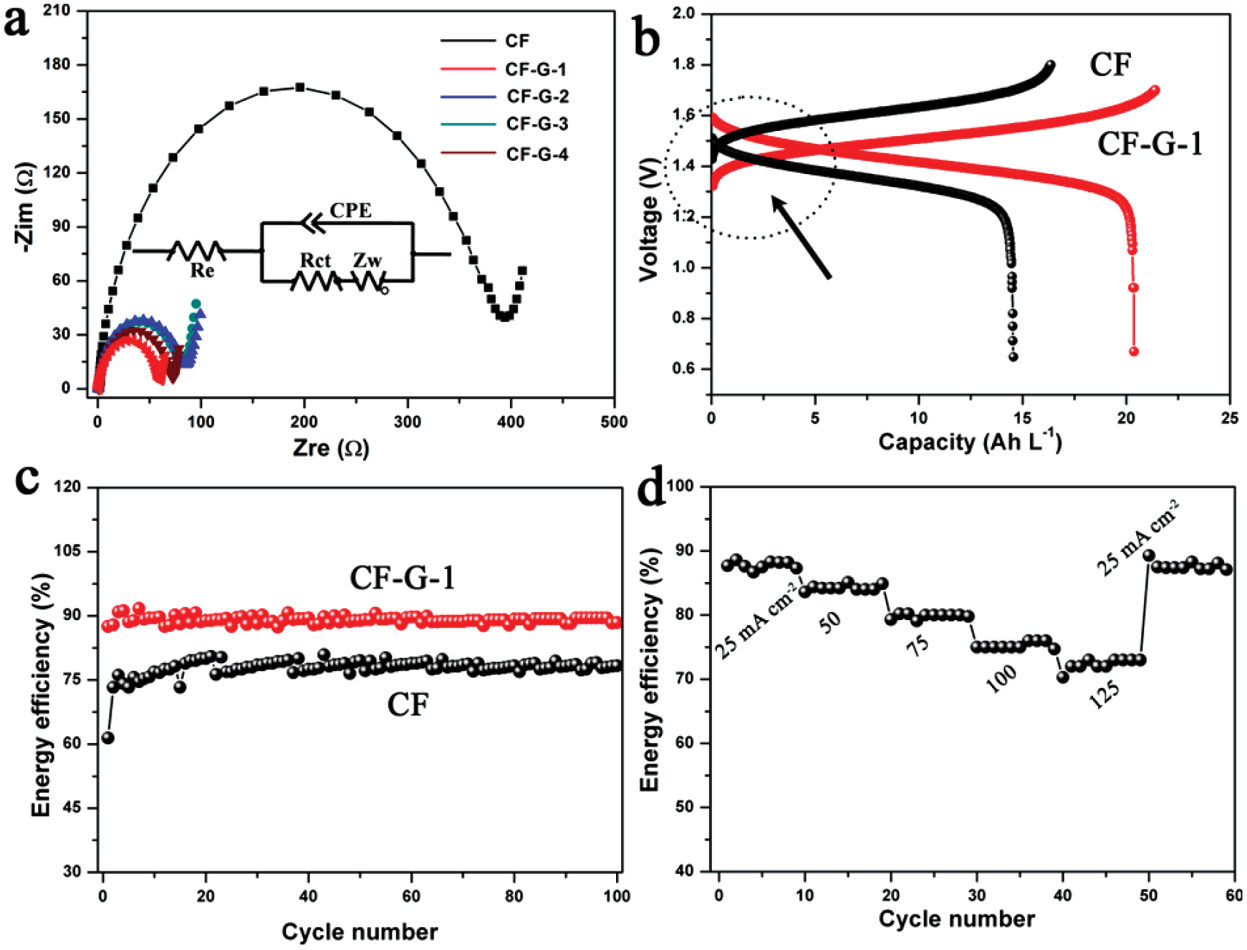
a) EIS plots for CF, CF‐G‐1, CF‐G‐2, CF‐G‐3, and CF‐G‐4 at open‐circuit potential, b) the voltage profiles for CF and CF‐G‐1 electrodes, c) energy efficiency values for CF and CF‐G‐1 electrodes within 100 cycles at the current density of 25 mA cm^−2^. d) Rating capability of VRFB with the CF‐G‐1 as positive electrode.

Electrochemical impendence spectroscopy (EIS) was used to measure the charge transfer resistance (Rct) during the redox reactions. As shown in **Figure** **5**a, all the EIS plots comprise one semicircle in the high‐frequency region and a straight line in the low‐frequency region.

The solution resistance (Re) and the Rct (summarized in Table S2, Supporting Information) can be respectively estimated from the starting point and the diameter of the semicircles. The resistance of diffusion (Zw) during the reaction process can be determined from the slope of the straight line.^41^, ^42^, ^43^ An equivalent circuit diagram of these electrodes is also shown in Figure 5a. The Rct value for the CF electrode is about 392 Ω, much larger than that for graphene‐nanowall‐modified CFs (e.g., the Rct value for CF‐G‐1 is only 55 Ω), suggesting the redox reactions can be accelerated significantly on the surface of the graphene nanowalls. As analyzed above, the CF‐G‐1 shows the highest electrochemical activity among these samples and was shown as electrode to test the battery performance. Charge–discharge voltage profiles of the VRFBs with CF‐G‐1 and CF are shown in Figure 5b. For CF‐G‐1 sample, the onset potential of charging branch is lower than that of CF, opposite behavior is observed for the discharging branch, implying the redox reactions on the CF‐G‐1 endures much smaller electrochemical polarization during the battery operation.^44^, ^45^, ^46^ As a result, more capacity can be obtained over the same operation potential window (0.7–1.7 V), about 20.5 Ah L^−1^ discharge capacity for CF‐G‐1 and 14.3 Ah L^−1^ discharge capacity for CF. For VRFB, the EE value is a key performance indicator for evaluating the energy conversion efficiency between chemical and electric energies. The energy efficiency (EE) within 100 cycles at the current density of 25 mA cm^−2^ for CF‐G‐1 and CF are shown in Figure 5c, the battery with a CF‐G‐1 electrode can still be maintained at ≈90% even after 100 cycles; while, the EE value for battery with a CF electrode is below 80%. More importantly, due to the dominant advantage on charge transportation afforded by the in situ grown graphene nanowalls, outstanding rate performance of ≈90%, ≈85%, 80%, 76%, and 73% EE values at current densities of 25, 50, 75, 100, and 125 mA cm^−2^ is obtained, respectively (Figure 5d). Remarkably, when the current density returns to 25 mA cm^−2^, the EE value reverts to ≈90%. This favorable high rate performance is supported by abundant specific surface area of the modified electrode and fast oxygen and electron transfer rate facilitated by the graphene nanowalls.^29^, ^35^, ^47^

To further test operation stability of the VRFB with a CF‐G‐1 electrode, we measured its capacity changes for 100 cycles at a current density of 25 mA cm^−2^, the capacity decreases from 20.5 Ah L^−1^ for the initial value to 9.7 Ah L^−1^ after 100 cycles (Figure S8, Supporting Information). As explain, degradation caused by the vanadium ion counter diffusion through the membrane is well‐known main cause for capacity fading in VRFB.^48^, ^49^, ^50^ To isolate its effects, we replace the membrane in the VRFB after 100 cycles without changing any other components. Capacity of the battery recovers to a value of 21.3 Ah L^−1^ which is larger than its initial value of 20.5 Ah L^−1^, indicating the present CF‐G‐1 electrode practically not only having no electrochemical degradation after 100 cycles, but also completing an activation process. The surface chemical status of CF‐G‐1 after 100 cycles was further analyzed by XPS method. As shown in Figure S9 of the Supporting Information, the surface oxygen content increases from 7.13% to 12.3%, suggesting some of the graphene (probably the edge part with more defects) has been oxidized in the strong oxidation electrolyte during the charge–discharge processes.^51^, ^52^, ^53^ The ratio between C—OH and O=C—OH functional groups for the fresh CF‐G‐1 is about 1:1 which increase to 2:1 after 100 cycles. These increased oxygen functional groups will provide more reactive sites for VO_2_^+^/VO^2+^ redox reactions. Inspired by this interesting phenomenon and previous reported activation method,^5^, ^8^, ^24^ we treated the CF‐G‐1 sample in concentrated nitric acid to introduce more surface oxygen functional groups and further evaluated as positive electrode for VRFB. As shown in Figure S10a–c of the Supporting Information, the oxygen atom percentage of CF‐G‐1 increases to 12.8% after the oxidation process which is even more than that of the CF‐G‐1 electrode after 100 cycles. The battery performance of the modified CF‐G‐1 is exhibited in Figure S10d of the Supporting Information, 21.9 Ah L^−1^ of initial discharge capacity can be obtained and the Coulombic efficiency keep above 96% at 50th cycle, further demonstrating that introducing of surface oxygen functional groups can improve the electrochemical activity of graphene toward VO_2_^+^/VO^2+^ redox reactions. The proposed catalytic mechanism is schemed in Figure S11 of the Supporting Information. For the oxidation process, VO^2+^ ions connect with the surface oxygen containing groups first, then the oxygen atom transfer from the H_2_O molecule to the VO^2+^ with the assistance of surface oxygen containing groups. Finally, the reaction product (VO_2_^+^) diffuses from the electrode surface into the electrolyte. For the reduction process, the reaction is opposite and can also be facilitated by the surface oxygen functional groups.^33^, ^35^ Due to the synergistic effect between the large specific surface area of graphene and abundant oxygen functional groups on them, robust battery performance can be achieved.

The structural stability is also crucial for stable cycling. The battery was disassembled and CF‐G‐1 electrode was washed and dried after battery test. SEM images for this used CF‐G‐1 electrode are shown in Figure S12a of the Supporting Information; the vertically standing characteristic of the graphene nanowall is well maintained without any sign of peeling off. Other than the electrode, operation stability of VRFB is also limited by the membrane which makes the battery couldn't operate for a long time. To eliminate the effects from this reason, we have specially designed a testing setup as shown in Figure S12b of the Supporting Information, the CF‐G‐1 electrode was putted into a glass container with flowing electrolyte (no membrane and current were applied during this adhesion strength test). After 10 d scouring test in the electrolyte, the CF‐G‐1 electrode was taken out and characterized by SEM (Figure S12c, Supporting Information). No obvious change can be observed and the graphene nanowalls still possess 3D structure and are well connect with the CF substrate, which further confirm that the bonding between carbon fibers and graphene nanowalls are strong enough to withstand the battery's mechanical operation.

## 3 Conclusion

In summary, graphene nanowall decorated CF was successfully fabricated via a simple MPCVD method and used as positive electrode for VRFB for the first time. Compared with previous reported graphene based catalysts, this unique structure can provide more active sites for vanadium redox reactions. Excellent cycle stability (the EE value can keep at ≈90% even after 100 cycles) and rate capability can be obtained when employing the composite materials as positive electrode during VRFB test. Moreover, this in situ growth process ensures good bonding between the graphene nanowalls and the carbon fibers. The electrode show no observable degradation in long time scouring test and 200 charge–discharge cycles. We believe this fabrication method and this novel structure will have a very good promise in VRFB electrode application. Moreover, this all carbon based material may also have applications in catalyst substrate and other battery electrodes.

### 4 Experimental Section

*Preparation of 3D Graphene Modified Carbon Felt Electrode*: The graphene nanowall decorated carbon felt was prepared by a simple MPCVD process, as reported elsewhere with modification.^54^, ^55^ Typically, dried carbon felt was cut into 2.5 × 2.5 cm and used as the substrates, which is placed on top of a molybdenum anode. The graphene nanowalls were grown on the carbon felt by a commercial 1.5 kW ASTeX MPCVD system. Plasma was ignited in hydrogen at 7 Torr. After the carbon felt was exposed to hydrogen plasma for 10 min to remove contamination, it is followed by a CH_4_/H_2_ gas mixture at a constant flow rate ratio at 30 Torr. The microwave power was kept at 1200 W, and the substrate was heated to about 800 °C as monitored by an infrared pyrometer and maintained for 2 h. After the plasma was switched off, the sample was cooled down naturally. Details on the growth conditions are listed in Table S1 of the Supporting Information. Samples synthesized under different conditions are marked as CF‐G‐1, CF‐G‐2, CF‐G‐3, and CF‐G‐4, respectively. CF‐G‐1 sample was further modified in concentrated nitric acid at 80 °C for 6 h, and then washed by deionized water and alcohol for several times and dried in vacuum.

*Characterization*: Structures of the materials were characterized with an X‐ray powder diffractometer (XRD, D8‐Advance, Bruker, Germany). Morphologies of the carbon felt and graphene‐modified carbon felts were observed with a transmission electron microscope (TEM, Philips CM‐200 FEG) at 200 kV and a scanning electron microscope (SEM, Philips XL30 FEG SEM). Raman spectroscopy was carried out with a Renishaw 2000 Raman microscope with 633 nm excitation source. Surface areas of the samples were measured with nitrogen sorption isotherms 77 K on a Micromeritics ASAP 2020 apparatus. The surface chemical status for CF‐G materials was analyzed by X‐ray photoelectron spectroscopy (XPS, VG Scientific ESCALab220i‐XL). Electric conductivity of all samples is tested by the equipment shown in Figure S3 and the test method is also described in detail in the Supporting Information.

*Electrochemical Measurement*: Electrochemical measurements were carried out using a three‐electrode cell on an electrochemical workstation (Autolab 302N). A working electrode with a geometric area of 1 cm^2^ was clamped by an electrode holder where only a small part of the carbon felt was contacted with the platinum current collector was equipped with a platinum plate counter electrode and Ag/AgCl reference electrode in 0.1 m VOSO_4_ (Sigma‐Aldrich, 99.5%) in 2 m H_2_SO_4_ solution. A salt bridge was used to eliminate the liquid junction potential. Cyclic voltammetry (CV) was recorded over a fixed voltage window between 0 and 1.4 V versus Ag/AgCl under different scan rates at room temperature. Electrochemical impendence spectroscopy (EIS) was recorded over a frequency range from 100 kHz to 10 mHz at open‐circuit voltage. For battery test, thickness of ≈4 mm graphene modified carbon felt electrode and pristine carbon felt (Liao Yang Carbon Fiber Sci‐tech. Co., Ltd. China) with an active area of 6.25 cm^2^ (2.5 × 2.5 cm) were used as the positive and negative electrode, respectively. The batteries were fabricated by sandwiching the proton exchange membrane (Nafion 212) between two pieces of electrodes placed in polytetrafluoroethylene frames. Copper sheet and conductive plastic were served as current collector and bipolar plate, respectively. Flexible graphite was place between the current collector and bipolar plate which was used to reduce the contact resistance during the battery operation due to the full contact between them. The carbon felt compression ratio in the battery apparatus is about 75%. The starting electrolytes for both the positive and the negative part were 2.5 m VOSO_4_+ 2.5 m H_2_SO_4_ (15 mL). The test batteries were charged and discharged over the operating potential range of 0.7–1.7 V.

## Supporting information

As a service to our authors and readers, this journal provides supporting information supplied by the authors. Such materials are peer reviewed and may be re‐organized for online delivery, but are not copy‐edited or typeset. Technical support issues arising from supporting information (other than missing files) should be addressed to the authors.

Supplementary

## References

[1] B. Dunn , H. Kamath , J. M. Tarascan , Science 2011, 334, 928. 22096188 10.1126/science.1212741

[2] A. Parasuraman , T. Lim , C. Menictas , M. Skyllas‐Kazacos , Electrochim. Acta 2013, 101, 27.

[3] W. Wang , Q. Luo , B. Li , X. Wei , L. Li , Z. Yang . Adv. Funct. Mater. 2013, 23, 970.

[4] Z. Yang , J. Zhang , M. C. Kintner‐Meyer , X. Lu , D. Choi , J. P. Lemmon , J. Liu , Chem. Rev. 2011, 111, 3577. 21375330 10.1021/cr100290v

[5] W. Li , J. Liu , C. Yan , Carbon 2013, 55, 313.

[6] S. Wang , X. Zhao , T. Cochell , A. Manthiram , J. Phys. Chem. Lett. 2012, 3, 2164. 26295765 10.1021/jz3008744

[7] C. Ding , H. Zhang , X. Li , T. Liu , F. Xiang , J. Phys. Chem. Lett. 2013, 4, 1281. 26282141 10.1021/jz4001032

[8] L. Yue , W. Li , F. Sun , L. Zhao , L. Xing , Carbon 2010, 48, 3079.

[9] M. Park , Y. Jung , J. Kim , H. Lee , J. Cho , Nano Lett. 2013, 13, 4833. 24024628 10.1021/nl402566s

[10] C. Flox , C. Fàbrega , T. Andreu , A. Morata , M. Skoumal , J. Rubio‐Garcia , J. R. Morante , RSC Adv. 2013, 3, 12056.

[11] M. Park , J Ryu , Y. Kim , J. Cho , Energy Environ. Sci. 2014, 7, 3727.

[12] Y. Shao , X. Wang , M. Engelhard , C. Wang , S. Dai , J. Liu , Z. Yang , Y. Lin , J. Power Sources 2010, 195, 4375.

[13] S. Zhong , M. Skyllas‐Kazacos , J. Power Sources 1992, 39, 1.

[14] M. Gattrell , J. Park , B. MacDougall , J. Apte , S. McCarthy , C. Wu , J. Electrochem. Soc. 2004, 151, A123.

[15] M. Chakrabarti , R. Dryfe , E. Roberts , Electrochimi. Acta 2007, 52, 2189.

[16] B. T. Sun , M. Skyllas‐Kazacos , Electrochim. Acta 1991, 36, 513.

[17] T. M. Tseng , R. H. Huang , C. Y. Huang , K. L. Hsueh , F. S. Shieu , J. Electrochem. Soc. 2013, 160, A690.

[18] K. J. Kim , M.‐S. Park , J.‐H. Kim , U. Hwang , N. J. Lee , G. Jeong , Y.‐J. Kim , Chem. Commun. 2012, 48, 5455. 10.1039/c2cc31433a 22540132

[19] C. Yao , H. Zhang , T. Liu , X. Li , Z. Liu , J. Power Sources 2012, 218, 455.

[20] Z. González , A. Sánchez , C. Blanco , M. Granda , R. Menéndez , R. Santamaría , Electrochem. Commun. 2011, 13, 1379.

[21] Z. He , L. Dai , S. Liu , L. Wang , C. Li , Electrochim. Acta 2015, 176, 1434.

[22] B. Li , M. Gu , Z. Nie , Y. Shao , Q. Luo , X. Wei , X. Li , J. Xiao , C. Wang , V. Sprenkle , W. Wang , Nano Lett. 2013, 13, 1330. 23398147 10.1021/nl400223v

[23] B. Li , M. Gu , Z. Nie , X. Wei , C. Wang , V. Sprenkle , W. Wang , Nano Lett. 2014, 14, 158. 24279888 10.1021/nl403674a

[24] P. Han , H. Wang , Z. Liu , X. Chen , W. Ma , J. Yao , Y. Zhu , G. Cui , Carbon 2011, 49, 693.

[25] W. Li , J. Liu , C. Yan , Carbon 2011, 49, 3463.

[26] J. Jin , X. Fu , Q. Liu , Y. Liu , Z. Wei , K. Niu , J. Zhang , ACS Nano 2013, 7, 4764. 23647240 10.1021/nn3046709

[27] Z. González , S. Vizireanu , G. Dinescu , C. Blanco , R. Santamaría , Nano Energy 2012, 1, 833.

[28] P. Han , Y. Yue , Z. Liu , W. Xu , L. Zhang , H. Xu , S. Dong , G. Cui , Energy Environ. Sci. 2011, 4, 4710.

[29] M. Park , I. Jeon , J. Ryu , J.‐B. Baek , J. Cho , Adv. Energy Mater. 2015, 5, 1401550.

[30] H.‐J. Peng , J.‐Q. Huang , M.‐Q. Zhao , Q. Zhang , X.‐B. Cheng , X.‐Y. Liu , W.‐Z. Qian , F. Wei , Adv. Funct. Mater. 2014, 24, 2772.

[31] U. Khan , A. O'Nell , M. Lotya , S. De , J. N. Coleman , Small 2010, 6, 864. 20209652 10.1002/smll.200902066

[32] Y. Huang , J. Liang , Y. Chen , Small 2012, 8, 1805. 22514114 10.1002/smll.201102635

[33] B. Sun , M. Skyllas‐Kazacos , Electrochim. Acta 1992, 37, 1253.

[34] L. Li , S. Kim , W. Wang , M. Vijayakumar , Z. Nie , B. Chen , J. Zhang , G. Xia , J. Hu , G. Graff , J. Liu , Z. Yang , Adv. Energy Mater. 2011, 1, 394.

[35] B. Sun , M. Skyllas‐Kazacos , Electrochim. Acta 1992, 37, 2459.

[36] S. Kim , M. Vijayakumar , W. Wang , J. Zhang , B. Chen , Z. Nie , F. Chen , J. Hu , L. Li , Z. Yang , Phys. Chem. Chem. Phys. 2011, 13, 18186. 21922094 10.1039/c1cp22638j

[37] H. Q. Zhu , Y. M. Zhang , L. Yue,W. S. Li , G. L. Li , D. Shu , H. Y. Chen , J. Power Sources 2008, 184, 637.

[38] R. H. Huang , C. H. Sun , T. M. Tseng , W. k. Chao , K. L. Hsueh , F. S. Shieu , J. Electrochem. Soc. 2012, 159, A1579.

[39] C. Flox , J. Rubio‐Garcia , R. Nafria , R. Zamani , M. Skoumal , T. Andreu , J. Arbiol , A. Cabot , J. R. Morante , Carbon 2012, 50, 2372.

[40] A. J. Bard , L. R. Faulkner , Electrochemical Methods Fundamentals and Applications, John Wiley and Sons, New York, NY, USA 2001.

[41] J. Xu , J. Shui , J. Wang , M. Wang , H.‐K. Liu , S. X. Dou , I.‐Y. Jeon , J.‐M. Seo , J.‐B. Baek , L. Dai , ACS Nano 2014, 8, 10920. 25290080 10.1021/nn5047585

[42] X. Cao , Y. Shi , W. Shi , X. Rui , Q. Yan , J. Kong , H. Zhang , Small 2013, 9, 3433. 23637090 10.1002/smll.201202697

[43] L. Zhang , G. Zhang , H. B. Wu , L. Yu , X. W. Lou , Adv. Mater. 2013, 25, 2589. 23553828 10.1002/adma.201300105

[44] K. Kim , S.‐W. Lee , T. Yim , J.‐G. Kim , J. W. Choi , J. Kim , M.‐S. Park , Y.‐J. Kim , Sci. Rep. 2014, 4, 6906. 25366060 10.1038/srep06906 PMC4219165

[45] G. Zhou , S. Pei , L. Li , D.‐W. Wang , S. Wang , K. Huang , L.‐C. Yin , F. Li , H.‐M. Cheng , Adv. Mater. 2014, 26, 625. 24458578 10.1002/adma.201302877

[46] G. Y. Zheng , Y. Yang , J. J. Cha , S. S. Hong , Y. Cui , Nano Lett. 2011, 11, 4462. 21916442 10.1021/nl2027684

[47] Y. Zhu , S. Murali , W. Cai , X. Li , J. W. Suk , J. R. Potts , R. S. Ruoff , Adv. Mater. 2010, 22, 3906. 20706983 10.1002/adma.201001068

[48] Z. Zhang , Z. Zhang , X. Li , Z. Mai , J. Zhang , Energy Environ. Sci. 2011, 4, 1676.

[49] M. J. Jung , J. Parrondo , C. G. Arges , V. Ramani , J. Mater. Chem. A 2013, 1, 10458.

[50] D. Chen , S Wang , M. Xiao , Y. Meng , Energy Environ. Sci. 2010, 3, 622.

[51] M. Yang , S. Moriyama , M. Higuchi , J. Nanosci. Nanotechnol. 2014, 14, 2974. 24734719 10.1166/jnn.2014.8578

[52] J. Zhang , H. Zou , Q. Qing , Y. Yang , Q. Li , Z. Liu , X. Guo , Z. Du , J. Phys. Chem. B 2003, 107, 3712.

[53] T. Ly , D. Duong , Q. Ta , F. Yao , Q. Vu , H. Jeong , S. Chae , Y. H. Lee , Adv. Funct. Mater. 2013, 23, 5183.

[54] J. Zhao , M. Shaygan , J. Eckert , M. Meyyappan , M. H. Rümmeli , Nano Lett. 2014, 14, 3064. 24784459 10.1021/nl501039c

[55] C.‐H. Tu , W. Chen , H.‐C. Fang , Y. Tzeng , C.‐P. Liu , Carbon 2013, 54, 234.

