# Supplementary material for: Graphene‐Nanowall‐Decorated Carbon Felt with Excellent Electrochemical Activity Toward VO2 +/VO2+ Couple for All Vanadium Redox Flow Battery
Source: Adv Sci (Weinh). 2015 Dec 31;3(4):1500276. doi: 10.1002/advs.201500276 (PMC5064734; doi:10.1002/advs.201500276)
Supplement: Supplementary file 1 — Supplementary [file ADVS-3-0p-s001.pdf]

Copyright WILEY-VCH Verlag GmbH & Co. KGaA, 69469 Weinheim, Germany, 2015.

ADVANCED SCIENCE
Open Access

Supporting Information

for *Adv. Sci.*, DOI: 10.1002/advs.201500276

Graphene-Nanowall-Decorated Carbon Felt with Excellent Electrochemical Activity Toward $VO_2^{\ +}/VO^{2+}$ Couple for All Vanadium Redox Flow Battery

Wenyue Li, Zhenyu Zhang, Yongbing Tang, Haidong Bian, Tsz-Wai Ng, Wenjun Zhang,* and Chun-Sing Lee**

Copyright WILEY-VCH Verlag GmbH & Co. KGaA, 69469 Weinheim, Germany, 2013.

Supporting Information

Graphene nanowalls decorated carbon felt with excellent electrochemical activity towards VO_2^+/VO^{2+} couple for all vanadium redox flow battery

Wenyue Li[12], Zhenyu Zhang[2], Yongbing Tang[1], Haidong Bian[2], Tsz-Wai Ng[2], Wenjun Zhang[2]*, Chun-Sing Lee[2]**

Table S1 Detailed informations about the growth conditions for graphene nanowall decorated carbon flet

Sample	Gas/sccm			Power/W	Pressure/Torr	Temperature/^{o}C	Time/min
	H_2	CH_4	N_2				
**CF-G-1**	100	40	10	1200	50	800	120
**CF-G-2**	100	40	10	1200	50	800	60
**CF-G-3**	100	40	0	1200	50	800	120
**CF-G-4**	50	50	0	1200	50	800	120

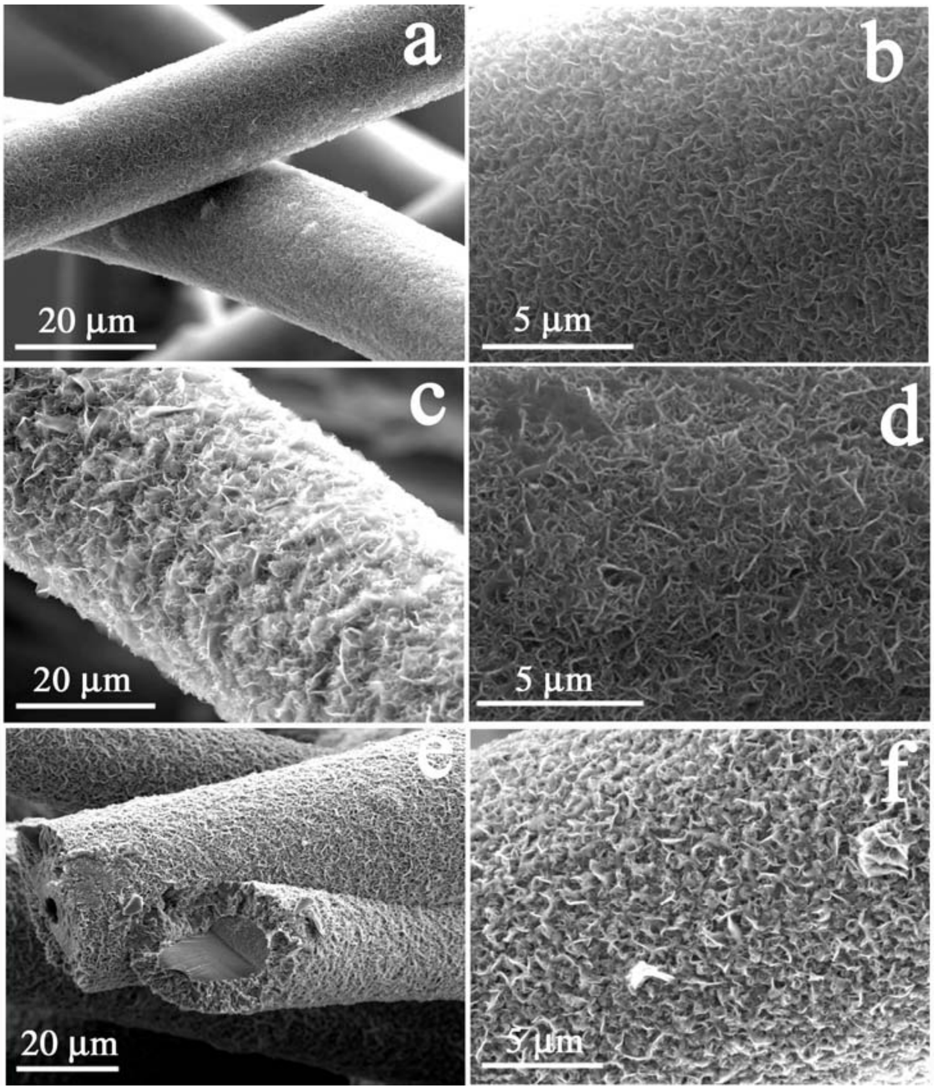

Figure S1 SEM images of CF-G-2 (a, b), CF-G-3 (c, d) and CF-G-4 (e, f) samples.

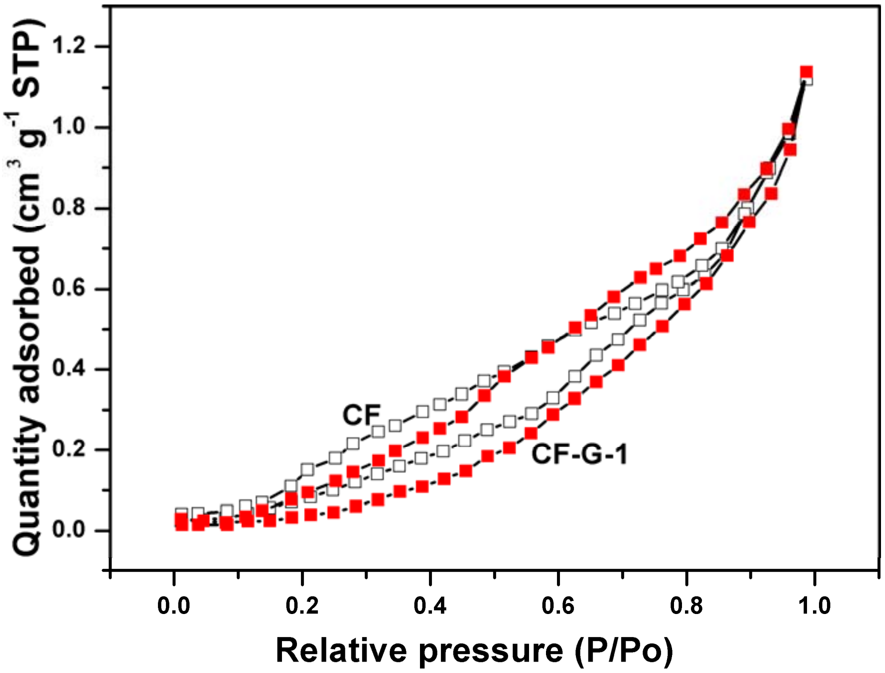

Figure S2 N_2 adsorption/desorption isotherms of CF and CF-G-1 samples (the specific surface area was determined by Brunauer-Emmett-Teller analysis)

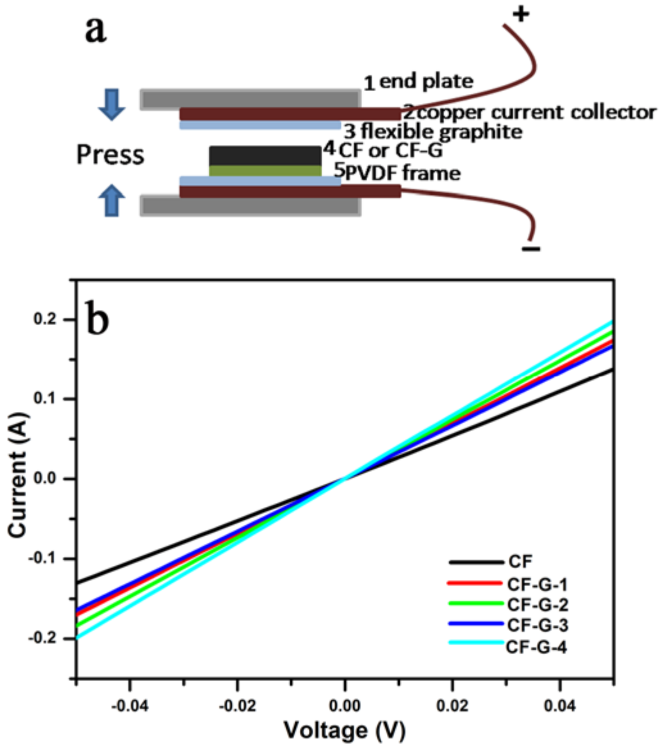

Figure S3 (a) Schematic diagram of area resistance test equipment, (b) I-V curves of different samples.

The area conductivity of these samples are tested by a equipment shown in Figure S3a. The sample with an area of 6.25 cm^2 was placed into the PVDF frame (2.5 * 2.5 cm) and pressed to 2 mm, then linear sweep voltammetry (LSV) technique was used to get the I-V curve (Figure 1b). Area resistance (R_s) of the sample can be calculated from the equation $R_s = (E/I-R_e)/S$, where E is the voltage, I is the current, R_e is the resistance of the equipment (~0.120 Ω) and S is the surface area of the sample. The R_s values of CF-G (20.5, 27.8, 23.9 and 26.0 mΩ cm^{-2} for CF-G-1, CF-G-2, CF-G-3 and CF-G-4, respectively) are smaller than that of the CF (36.4 mΩ cm^{-2}), demonstrating the enhanced conductivity of the CF-G electrodes.

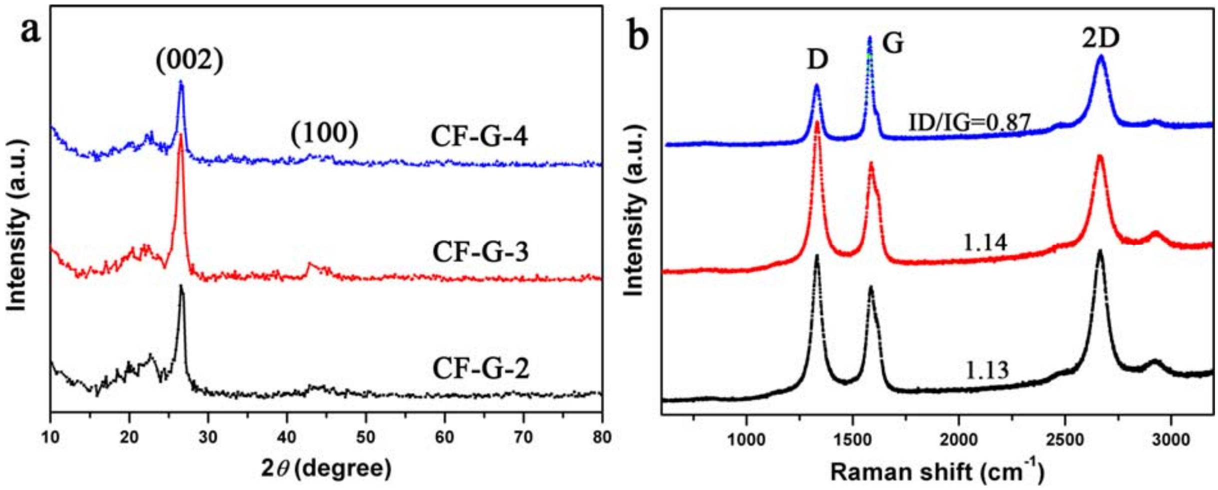

Figure S4 XRD patterns (a) and Raman spectra (b) for CF-G-2, CF-G-3 and CF-G-4.

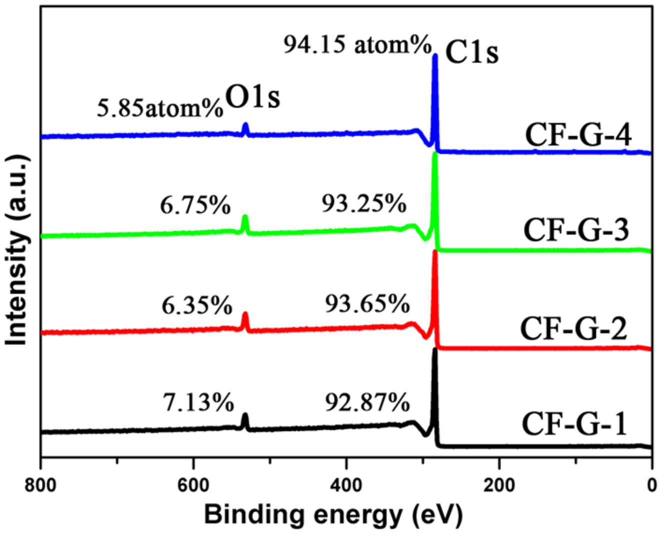

Figure S5 XPS survey spectra and element percentage for CF-G materials.

Table S2 Electrochemical properties obtained from the cyclic voltammetry curves at the scan rate of 5 mV s^{-1} and EIS plots for different electrodes.

	I_{pa} (A)	$-I_{pc}$ (A)	E_{pa} (V)	E_{pc} (V)	$I_{pa}/-I_{pc}$	ΔE (V)	R_{ct} (Ω)
**CF**	0.0126	0.0062	1.150	0.593	2.032	0.557	392.4
**CF-G-1**	0.0425	0.0299	1.061	0.692	1.421	0.369	55.3
**CF-G-2**	0.0343	0.0197	1.069	0.675	1.741	0.394	84.2
**CF-G-3**	0.0424	0.0266	1.041	0.702	1.594	0.339	85.6
**CF-G-4**	0.0392	0.0311	1.047	0.708	1.260	0.339	72.4

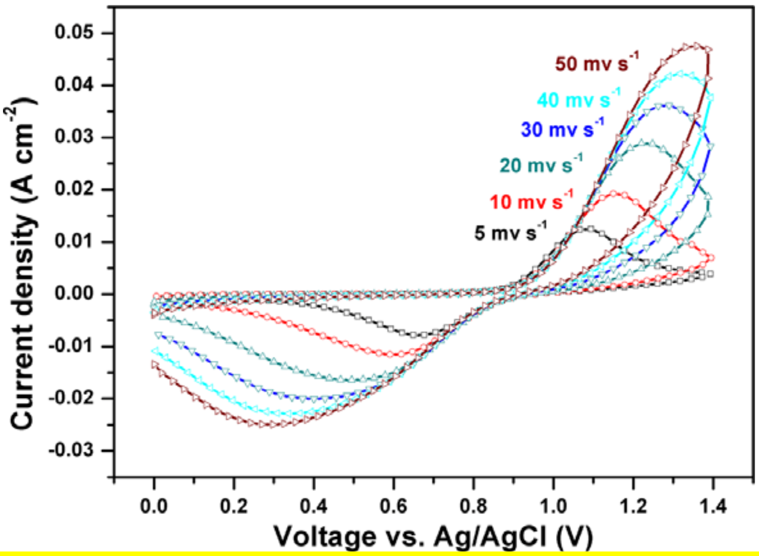

Figure S6 CV curves of CF electrodes under different scan rates range from 5 mv s^{-1} to 50 mv s^{-1}.

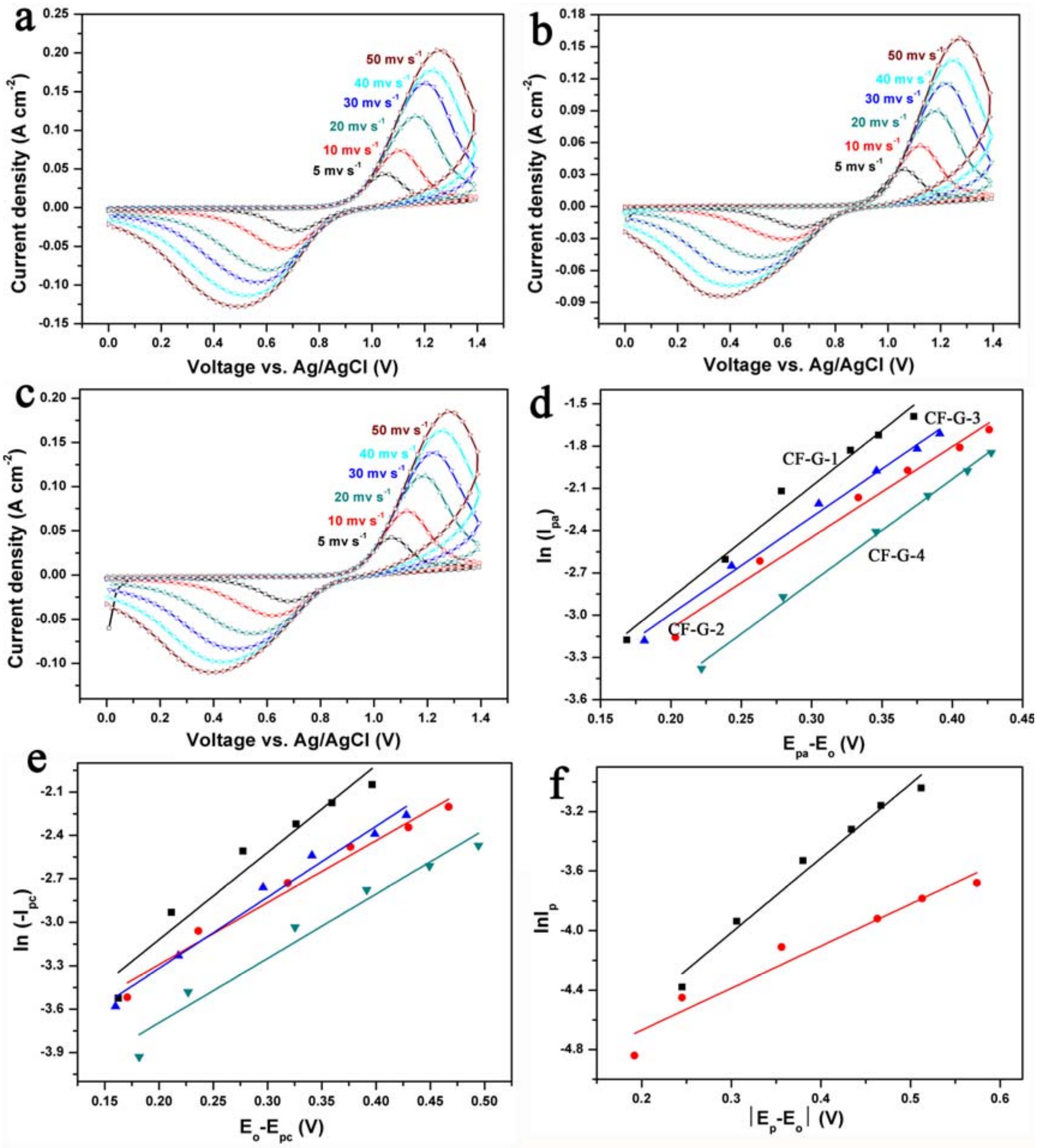

Figure S7 CV curves of CF-G-2 (a), CF-G-3 (b) and CF-G-4 (c) electrodes under different scan rates range from 5 mv s^{-1} to 50 mv s^{-1}. The fitting lines of $\ln|Ip|$ and $|(Ep-Eo)|$ for VO^{2+} oxidation (d) and VO_2^+ reduction (e) processes on different electrodes. The fitting lines of $\ln|Ip|$ and $|(Ep-Eo)|$ for VO^{2+} oxidation (a) and VO_2^+ reduction processes on the CF electrode (f).

$$Eo = \frac{\sum_{i=1}^{m}(Epai + Epci)}{2m} \qquad \textbf{(S1)}$$

where *Eo* is the equilibrium potential, *Epa* is anodic peak potential, *Epc* is the cathodic peak potential, *m* is the total number of peaks.

The reversibility of the reaction can be estimated by the value of $|(Epa-Epc)|$/n (n is the tansfer electron numer during oxidation/reduction processes). The reaction is irreversible if

the value is larger than 57~63, as shown in figure S6, the values of $|(Epa - Epc)|$/n (~300-800 mV varying with scan rate) are much larger than 57~63, suggesting the reaction system in our experiment is totally irreverbile. The following equation can be utilized to calculate reaction rate constant of totally irreversible reactions.

$$Ip = 0.227 nFAC_o^* K^o \exp(-\alpha f(Ep - Eo)) \qquad \textbf{(S2)}$$

Where *Ip* is the peak current density, *n* is number of transfer electron during electrode reaction (n = 1), *F* is faraday constant (F = 96485 C/mol), *A* is surface area of the electrode (geometric area ~ 1 cm^2), C_o^* is the electrolyte concentration (0.1 M), K^o is the reaction rate constant, α is the symmetry coefficient and *Ep* is peak potential. A plot of $\ln|Ip|$ *vs.* $|(Ep - Eo)|$ determined at different scan rates have a slope of $-\alpha f$ and an intercept proportional to K^o. The K^o can be obtained from the intercept without using the α value.

Table S3 The fitting result of Intercepts and reaction rate constants of VO^{2+} oxidation and VO_2^+ reduction processes on different electrodes.

Sample	Intercept(anodic)	K_a^o (cm s^{-1})	Intercept(cathodic)	K_c^o (cm s^{-1})
**CF**	-5.515	1.84×10^{-3}	-5.234	2.43×10^{-3}
**CF-G-1**	-4.367	5.79×10^{-3}	-4.149	7.20×10^{-3}
**CF-G-2**	−4.960	3.20×10^{-3}	−5.70	4.67×10^{-3}
**CF-G-3**	-4.383	5.70×10^{-3}	-4.299	6.20×10^{-3}
**CF-G-4**	-4.463	5.26×10^{-3}	-4.323	6.05×10^{-3}

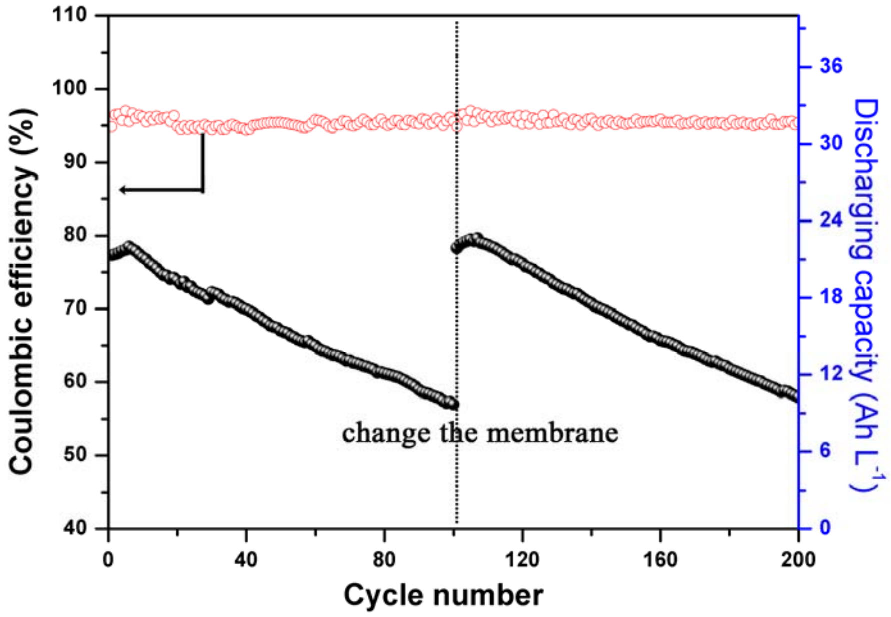

Figure S8 The VRFB performance with the CF-G-1 as positive electrode within 200 cycles (change the membrane after 100 cycles).

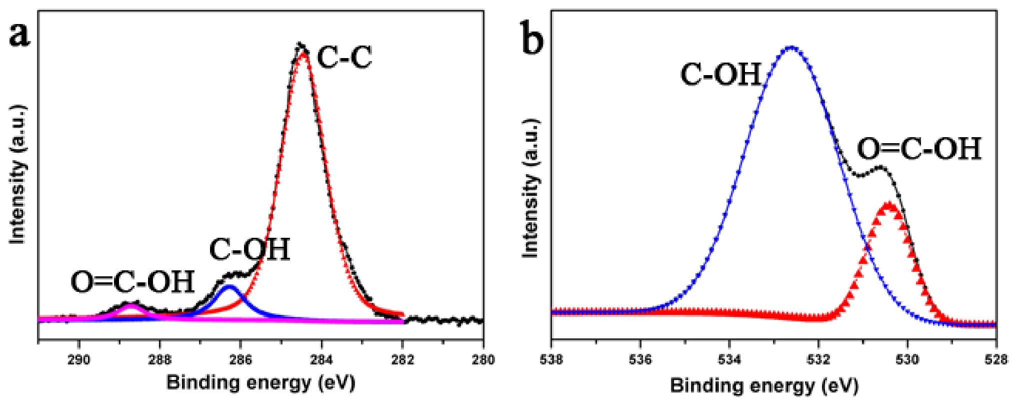

Figure S9 XPS C1s and O1s high-resolution spectra for CF-G-1 after 100 cycles.

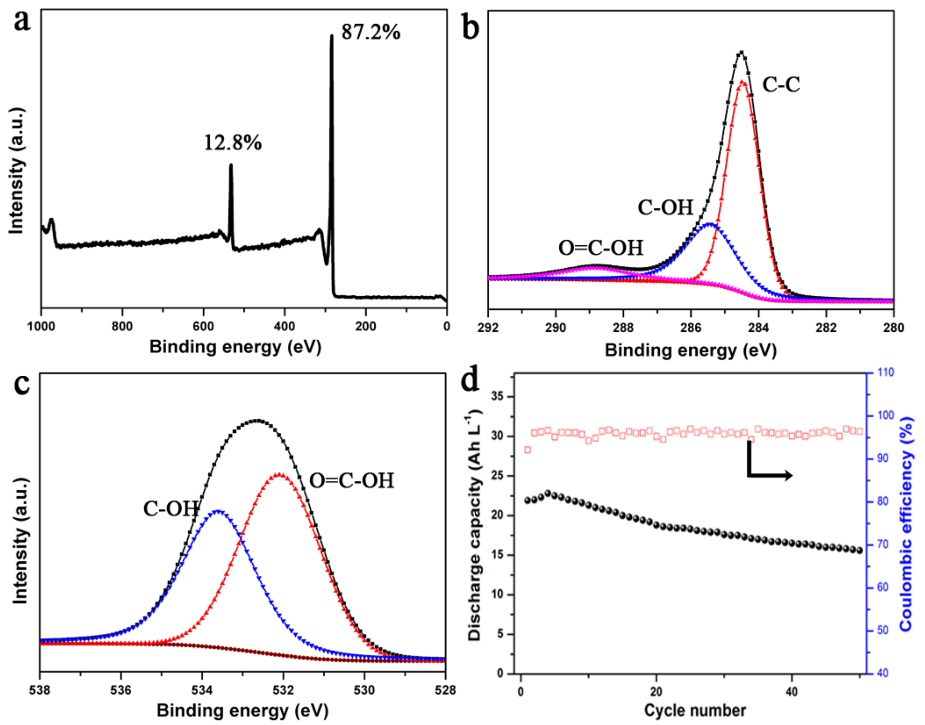

Figure S10 XPS survey (a), C1s high-resolution (b) and O1s high-resolution spectra (c), and VRFB performance of CF-G-1 of after concentrated nitric acid treatment (d).

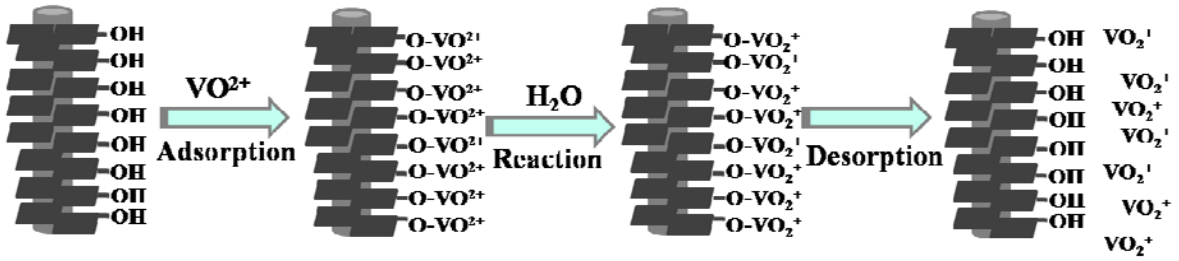

Figure S11 The proposed catalytic mechanism for CF-G materials during VO_2^+/VO^{2+} redox reactions.

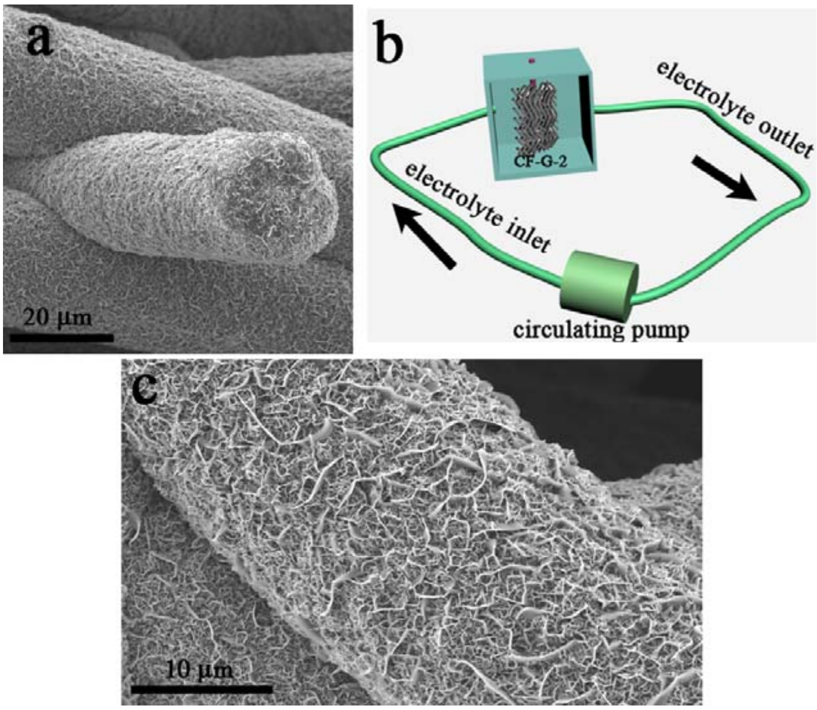

Figure S12 SEM images for CF-G-1 after 200 cycles (a) Homemade electrode stability test apparatus (b), SEM image after long structure stability test (c).
